# Predictors of functional independence, quality of life, and return to work in patients with burn injuries in mainland China

**DOI:** 10.1186/s41038-016-0058-4

**Published:** 2016-11-04

**Authors:** Dan Tang, Cecilia W. P. Li-Tsang, Ricky K. C. Au, Xia Shen, Kui-cheng Li, Xian-feng Yi, Lin-rong Liao, Hai-yan Cao, Ya-nan Feng, Chuan-shun Liu

**Affiliations:** 1 Department of Rehabilitation Sciences, The Hong Kong Polytechnic University, Hung Hom, Hong Kong Special Administrative Region China; 2 Guangdong Provincial Work Injury Rehabilitation Hospital, Guangzhou, China; 3 Department of Physiotherapy, Guangdong Provincial Work Injury Rehabilitation Hospital, Guangzhou, China; 4 Department of Occupational Therapy, Guangdong Provincial Work Injury Rehabilitation Hospital, Guangzhou, China; 5 Department of Burn Surgery and Burn Rehabilitation, Guangdong Provincial Work Injury Rehabilitation Hospital, Guangzhou, China

**Keywords:** Burns, Functional independence, Quality of life, Return to work, Predictors, Rehabilitation

## Abstract

**Background:**

Burn injury may be associated with long-term rehabilitation and disability, while research studies on the functional performance after injuries, quality of life (QOL), and abilities to return to work of burn patients are limited. These outcomes are related not just to the degree and nature of injuries, but also to the socio-economical background of the society. This study aimed to identify the factors which might affect burn patients’ abilities to reintegrate back to the society based on a sample in mainland China.

**Methods:**

A retrospective study was conducted to collect data of demographic characteristics, medical data about burn injuries, physical and psychological status, and self-perceived QOL at the initial phase and upon discharge from a rehabilitation hospital, timing of rehabilitation, and duration of rehabilitation intervention. Four hundred fifteen patients with burn injuries were recruited in the study. Multiple linear regression and logistic regression were used to obtain a model to predict the functional abilities and the perceived QOL at discharge and their changes during rehabilitation, as well as the post-injury work status within 6 months after discharge.

**Results:**

The functional performance at discharge and its change were significantly predicted by the functional abilities and QOL at the admission, duration of treatment, timing of rehabilitation, payer source, and total body surface area burned. The perceived QOL at discharge and its change were significantly predicted by the baseline QOL at admission and duration of treatment. The significant predictors of work status within 6 months post-discharge included age, education, payer source, total body surface area burned, perceived QOL, and bodily pain at admission.

**Conclusions:**

The present study identified a number of factors affecting the rehabilitation outcomes of people with burn injuries. Identification of these predictors may help clinicians assess the rehabilitation potential of burn survivors and assist in resource allocation. Policy makers should ensure that resources are adequate to improve the outcomes based on these factors.

## Background

Burn injury is a devastating form of injury and also a major global public health issue. There are about 900 inpatients per burn center annually in 39 burn centers located in 17 provinces in mainland China. Nevertheless, there are only limited epidemiological data on burn injuries in mainland China, and it has been reported that the incidence rate of burn injuries peaks in individuals at the working age [1]. Burn injuries could affect patients’ functional abilities, reduce quality of life (QOL), and impede their abilities to return to work (RTW) [2]. Therefore, it is essential to explore factors which affect these outcomes in burn rehabilitation, which as a result, may help optimizing rehabilitation services in this population.

There are a few studies exploring the predictors of post-burn functional independence, QOL and work status, and gains in these outcomes from burn rehabilitation [3–5]. However, most of these studies were conducted in the developed countries, where the incidence rate [6], demographic patterns of burns [7], health care financing, and delivery systems [8] are largely different from those in the developing countries. Burns are much more common in the developing countries than in the developed regions due to poverty, substandard living conditions, overcrowding, and illiteracy [6]. As reported by the WHO [9], the majority of burn-related deaths occur in the developing countries, particularly in South-East Asia. Among the burn-injured patients, the male-to-female ratio and the average age is higher in the developed countries than in the developing regions [7]. In addition, the medical security coverage and the accessibility of burn care including rehabilitation treatments are much better in the developed countries than in the developing ones [8, 10]. These differences can result in dissimilar impacts on post-burn functional independence, QOL, and work status in people receiving burn rehabilitation. Therefore, the research objective of the present study was to identify the predictors of functional independence, QOL, and post-burn work status in burn rehabilitation patients based on a population sample in mainland China.

## Methods

### Study design and subject selection criteria

The present study employed a longitudinal retrospective design. The data were obtained by the Department of Burn Rehabilitation of the Guangdong Provincial Work Injury Rehabilitation Hospital from January 2009 to December 2014. All patients were referred by the burn surgeons in the acute hospital in the region. Patients admitted to the rehabilitation hospital would receive a multidisciplinary rehabilitation program including passive and active exercises, splinting and positioning, management of edema and pain, training of activities of daily living (ADL), education of patients and families, and psychosocial support. The program was implemented daily for 6 h excluding Sunday. Subject recruited in this study should have a diagnosis of burn injuries, first admission to the hospital, and between 18 and 60 years old. Those patients with multiple admissions to the hospital or had received other therapy prior to admission were excluded from the study. Those with severe complications unrelated to their burn injuries which impaired functional independence, QOL, or work capacity, such as traumatic brain injuries, spinal cord injuries, serious fractures, amputations, or severe infection would be excluded as well. The study was approved by the Human Research Ethics Committee of the Guangdong Provincial Work Injury Rehabilitation Hospital (no. AF/SC-07/2013.01) and The Hong Kong Polytechnic University (no. HSEARS20150708003).

### Factors affecting the QOL and RTW status of patients with burn injuries

The evaluation was measured at admission to the rehabilitation hospital and upon discharge, and at 6 months after discharge (follow-up).

The Chinese version of the Modified Barthel Index (MBI) comprises 10 scored activities including personal hygiene, bathing, dressing, feeding, bowel control, bladder control, toilet transfers, stair climbing, wheelchair/chair-bed transfer, and ambulation [11]. Each activity is scored on a 5-point ordinal scale which varies from item to item (i.e., 0, 1, 3, 4, or 5 for personal hygiene and bathing; 0, 2, 5, 8, or 10 for dressing, bowel control, bladder control, toilet transfer, and stair climbing; and 0, 3, 8, 12, or 15 for wheelchair/chair-bed transfer, and ambulation). A total score of 75 to 95 indicates mild functional dependence, and a score of 100 implies complete functional independence.

The World Health Organization Quality of Life (WHOQOL)-BREF scale is a generic measure of QOL, including four domains (physical health, psychological health, social relationships, and environment) [12]. The WHOQOL-BREF comprises four subscales with 28 items that measure different domains of QOL. Each item is rated from 1 (very poor/very dissatisfied/not at all) to 5 (very good/very satisfied/completely satisfied). The score of each domain is transformed into a scale of 0 to 100 to enable comparisons between domains composed of unequal numbers of items. Information on subjects’ working status before admission was collected at the time of admission and was further followed up by social workers via telephone during the 6 months after discharge.

The demographic characteristics, medical data about burn injuries, physical and psychological status which were recorded at admission, the time from injury till rehabilitation, and the duration of the rehabilitation (days) were also recorded. The demographic characteristics included age, gender, marital status, level of education, type of payer source for the medical care service (work injury insurance, medical insurance, or unreimbursed), self-perceived economic status, and family and company support were measured using a questionnaire, with the response graded as good, fair, and bad. The medical conditions, including the causes of injuries, burnt area, depth, and regions of burns were also documented. The physical status at admission included MBI (which reflects functional independence), pain and itch (which were measured by the visual analogue scale (VAS)), the number of joints with limited range of motion, and the Pittsburgh Sleep Quality Index (PSQI) were measured. The VAS is a tool used to describe a subjective quality such as pain [13] and itch [14], and is widely used in individuals with burns [13, 14]. For pain and itch, the subjects were asked to rate their worst pain levels once using the 100-mm VAS, with the far left of the scale indicating “no pain/itch” and the far right of the scale indicating “the worst pain/itch possible”. The PSQI is a self-rating questionnaire with a good test-retest reliability that provides an index of sleep quality for a 1-month interval [15]. It includes seven components with 19 items. A global sleep quality index is computed from the seven components which are scored with a 4-point ordinal scale from 0 to 3. The total score of PSQI ranges from 0 (indicating no difficulty at all) to 21 (indicating extreme difficulty).

The psychological status was measured by the self-rating depression and anxiety scales (SDS and SAS) [16]. The SDS is a valid and reliable 20-item measure, which rates the affective, psychological, and somatic symptoms associated with depression [17]. The subjects indicated how often they experience each symptom on a 4-point ordinal scale from 1 (a little of the time) to 4 (most of the time). The total score of SAS ranges from 20 to 80 with higher score indicating greater depression [17]. The SAS is also a 20-item self-administrated measure, which assesses somatic symptoms associated with anxiety. The scoring criteria and interpretation of the total score of the SAS are the same as the SDS [18]. The timing of rehabilitation was calculated from the onset of burn injury to the time the patient started the rehabilitation program. The duration of the rehabilitation was counted from the date of admission till the date of discharge.

### Statistical analysis

The collected data were numerically coded and analyzed using the SPSS Statistics software (version 19.0, Armonk, NY, USA). The demographic information of the subjects and burn injury characteristics were summarized with descriptive statistics. Paired *t* tests and chi-square tests were used to explore the changes on MBI, WHOQOL-BREF, and RTW in the subjects. The strength of association between the predicting variables and the outcome measures at discharge, as well as the change in outcome measures from admission, were determined by the Spearman’s correlation coefficient. The predictor variables which showed significant associations with the outcome measures were entered into multiple linear regression (for MBI and WHOQOL-BREF) and logistic regression analyses (for RTW) to identify the key predictors for the rehabilitation outcomes. For RTW, the total body surface area (TBSA) burned, which has been identified as a significant predictor in previous studies [19, 20], was controlled for when the effects of other predictors on RTW were explored. The level of significance was set at 5 % (two-tailed).

Among the data we collected, some cases were found to have missing data in some measurements. Thus, we considered an imputation procedure to evaluate the effect after excluding these cases from further analysis. Most of the missing data were the subjects’ socio-demographic data. Therefore, only age, education, expenditure, economic status, family support, employer support, rehabilitation days, and duration of treatment were imputed, and were treated as continuous variables (Table 1). These missing data showed a non-monotone missing pattern, so multiple imputation procedure with expectation-maximization (EM) algorithm was used to impute the missing data. EM algorithm is an iterative method for finding maximum likelihood or maximum a posteriori estimates of parameters in statistical models, where the model depends on unobserved latent variables. The results showed that the relative efficiency of all variables in Table 1 were greater than 0.99, justifying the imputations. These cases were therefore excluded from further analysis.

**Table 1:** Multiple imputation variance

Variable	Mean	Standard error	Total variance	Degree of freedom	Relative increase in variance	Fraction missing information	Relative efficiency
Age	34.940	0.480	0.230	440.080	0.007	0.007	0.999
Education	2.440	0.041	0.002	435.650	0.010	0.010	0.998
Expenditure	1.537	0.040	0.002	441.790	0.005	0.005	0.999
Economic status	2.060	0.025	0.001	441.790	0.005	0.005	0.999
Family support	2.606	0.028	0.001	443.620	0.002	0.002	1.000
Employer support	2.361	0.030	0.001	438.250	0.008	0.008	0.998
Rehabilitation days	5.406	0.277	0.077	385.910	0.034	0.033	0.993
Duration of treatment	131.583	4.381	19.197	429.550	0.014	0.014	0.997

## Results

### Sample characteristics

**Fig. 1:**
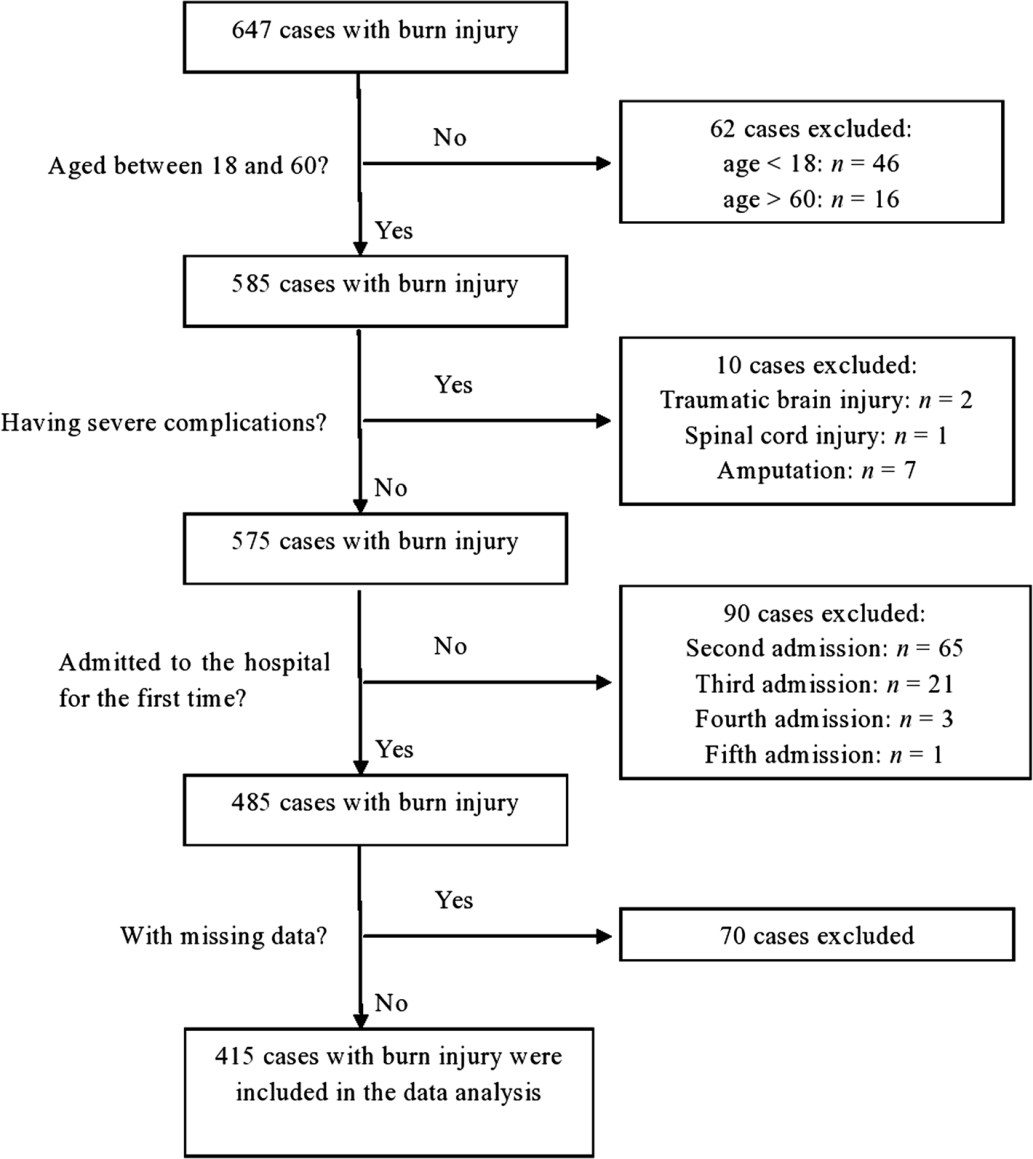
The flow chart of the case screening procedure

Six hundred forty-seven patients with burn injury were admitted to the hospital for rehabilitation during the period from 2009 to 2014. These cases were screened through the selection criteria. Five hundred eighty-five patients who were between 18 and 60 years old were selected. Among the 585 cases, 10 cases had severe complications, 90 were admitted to the hospital for more than one time, and 70 had missing data (Table 2). As a result, a total of 415 cases were included in the data analysis. Figure 1 shows a flow chart of the case screening procedure. Among the 415 cases, majority were male, with a mean age of 34.6 years and the mean time of starting rehabilitation was 157 days post-burn injuries. The demographic data and the nature of burn injuries are presented in Table 3. Most demographic data and the nature of burn injuries were comparable between those with and without missing data, except for the payer source and the duration of rehabilitation. Those cases with missing data were those who paid their own expenses for rehabilitation treatment and had relatively shorter period of rehabilitation (*p* < 0.05) (Table 3).

**Table 2:** Variables with missing data and the number of missing data in each variable

Variable	No. of missing data
Expenditure	2
Family support	2
MBI at admission	10
WHOQOL-BREF at admission	15
Number of joints with limited ROM at admission	50
MBI at discharge	8
WHOQOL-BREF at discharge	12
Number of joints with limited ROM at discharge	18
Work status after discharge	4

*MBI* Modified Barthel Index, *ROM* range of motion, *WHOQOL-BREF* World Health Organization Quality of Life BREF

**Table 3:** Demographic and medical characteristics of the study population

	Cases included (*n* = 415)	Cases with missing data (*n* = 70)	*p* value
Demographic characteristics			
Gender			0.055
1: Male	357 (86 %)	66 (94 %)	
0: Female	58 (14 %)	4 (6 %)	
Age: mean (SD)	34.6 ± 9.9		
Marital status			0.483
1: Married	290 (70 %)	52 (74 %)	
0: Single	125 (30 %)	18 (26 %)	
Education			0.110
1: Primary school	54 (13 %)	5 (7 %)	
2: Junior middle school	168 (40 %)	27 (37 %)	
3: High school	149 (36 %)	31 (44 %)	
4: University	42 (10 %)	5 (7 %)	
5: Higher levels	2 (1 %)	2 (3 %)	
Expenditure			< 0.001
Work injury insurance	294 (70 %)	31 (44 %)	
Medical insurance	27 (7 %)	6 (9 %)	
Own expense	94 (23 %)	31 (44 %)	
Economic status			0.616
1: Good	48 (12 %)	11 (16 %)	
2: Fair	297 (72 %)	48 (69 %)	
3: Bad	70 (16 %)	11 (16 %)	
Family support			0.099
1: Good	25 (6 %)	8 (11 %)	
2: Fair	117 (28 %)	23 (33 %)	
3: Bad	273 (66 %)	37 (53 %)	
Employer support			0.580
1: Good	35 (9 %)	10 (14 %)	
2: Fair	192 (46 %)	38 (54 %)	
3: Bad	188 (45 %)	22 (31 %)	
Burn characteristics			
Cause			0.521
Fire	212 (51 %)	37 (53 %)	
Thermal	74 (18 %)	14 (20 %)	
Blast	40 (10 %)	8 (11 %)	
Electrical	54 (13 %)	4 (6 %)	
Others	35 (8 %)	7 (10 %)	
Area			0.213
1: <30 %	145 (35 %)	17 (24 %)	
2: 30–50 %	93 (22 %)	14 (20 %)	
3: 50–90 %	127 (31 %)	28 (40 %)	
4: >90 %	50 (12 %)	11 (16 %)	
Depth			0.249
1: Epithelium	126 (30 %)	16 (22 %)	
2: Top aspect of the dermis	247 (60 %)	49 (70 %)	
3: Dermis	42 (10 %)	5 (7 %)	
Region			0.337
Above neck	5 (1 %)	0 (0 %)	
Upper limbs	32 (8 %)	3 (4 %)	
Lower limbs	18 (4 %)	5 (7 %)	
Trunk	4 (1 %)	2 (3 %)	
Multiple regions	356 (86 %)	60 (86 %)	
Rehabilitation days since onset: median (IQR)	109 (60–181)	90 (60–180)	0.330
Duration of treatment (days): median (IQR)	90 (60–180)	60 (30–127)	< 0.001

*IQR* interquartile range, ​*SD* standard deviation

### MBI, WHOQOL-BREF, and RTW after burn injuries

Table 4 shows the MBI, WHOQOL-BREF, and status of RTW before and after the rehabilitation program and the changes between the two intervals. At admission, the subjects showed mild functional dependence with a mean MBI score of 80.6, and significantly improvement was shown after rehabilitation with a mean increase of 14.2 (*p* < 0.001). For QOL, the WHOQOL-BREF scores were found to be increased after rehabilitation as well (*p* < 0.001). Three subjects had already returned to work before admission, and the reason for admission was on enhancement of outcomes. Within 6 months after discharge, 61 % (253 out of 415) of the subjects had returned to work. The ratio of people who did and did not RTW increased significantly over the course of rehabilitation (*p* < 0.001).

**Table 4:** Rehabilitation outcomes after burn injuries

	MBI score^a^ (mean ± SD)	WHOQOL-BREF^a^ (mean ± SD)				RTW (Y/N)^b^
		Physical health	Psychological health	Social relationships	Environment	
At admission	80.6 ± 21.5	42.6 ± 15.5	53.7 ± 12.9	48.8 ± 14.8	56.0 ± 13.0	3/412
At discharge	94.9 ± 10.9	53.5 ± 13.7	57.8 ± 12.7	54.8 ± 14.7	59.1 ± 13.8	253/162 (within 6 months after discharge)
Changes during inpatient period	14.2 ± 16.4	11.1 ± 13.6	7.2 ± 12.0	4.3 ± 11.8	4.3 ± 10.9	
*p* value for rehabilitation effects	< 0.001	< 0.001	< 0.001	< 0.001	< 0.001	< 0.001
Effect size	0.48	0.25	0.34	0.41	0.24	0.67

*MBI* Modified Barthel Index, *WHOQOL-BREF* World Health Organization Quality of Life BREF, *RTW* return to work, *SD* standard deviation

^a^Paired *t* test

^b^Chi-square test

### Prediction of outcomes and their changes upon discharge

The outcomes at discharge and their changes from admission showed significant correlations with different variables (Table 5). Those with strong association with the outcomes were then selected for regression analysis. Prior to conducting multivariate regression analysis, the intercorrelations between these predictor variables were calculated as a measure of multicollinearity that biases the estimation of regression coefficients. The correlations among PSQI, SDS, and SAS were high (*r* = 0.773 to 0.928). Therefore, only SDS was chosen to be included in the regression analysis. The highest inter-correlation between the remaining predictor variables was *r* = 0.698, between the scores of physical and psychological domains of WHOQOL-BREF scale, suggesting that multicollinearity was not a problem for the sample.

**Table 5:** Correlational analysis between the predictor variables and the outcomes at discharge and the change during the inpatient period of the subjects

	At discharge					Change from the baseline					RTW (1:Y, 0: N) within 6 months after discharge
	MBI	QOL1	QOL2	QOL3	QOL4	MBI	QOL1	QOL2	QOL3	QOL4	
Demographic characteristics											
Gender	.025	−.043	−.006	−.019	−.067	−.020	−.071	−.083	−.002	−.078	.105*
Age	−.113*	−.139*	−.121*	−.078	−.085	.067	.087	.075	.047	.012	−.185*
Marriage	.071	.105*	.027	.023	.027	−.029	−.108*	−.080	−.034	−.020	.149*
Education	.032	−.017	.031	−.028	−.024	.003	−.076	−.046	−.034	−.052	.178*
Payer source	−.415	−.157*	−.112*	−.147*	−.070	.138*	.058	.048	−.039	.000	−.250*
Economic status	−.069	.035	.080	.086	.070	−.002	−.028	−.002	.050	−.022	.068
Family support	.062	.104*	.072	.150*	.053	−.173*	−.014	.014	.039	−.028	.078
Company support	.137*	.122*	.118*	.158*	.105*	−.067	−.001	−.003	−.019	.058	.094
Burn characteristics											
Cause	.084	−.006	.135*	−.010	.025	−.088	−.041	−.002	−.073	−.047	−.009
Area	−.489*	−.292*	−.291*	−.212*	−.257*	.333*	.201*	.243*	.154*	.094	−.340*
Depth	−.138*	−.031	−.067	.060	−.076	.018	.083	.024	.045	.023	−.098*
Regions	−.180*	−.055	−.101*	−.049	−.071	.152*	.109*	.120*	.084	.090	−.135*
Physical, psychological characteristics at admission											
MBI score	.587*	.154*	.107*	.184*	.043	−.912*	−.325*	−.310*	−.184*	−.158*	.172*
QOL1	.387*	.560*	.443*	.414*	.409*	−.322*	−.570*	−.333*	−.221*	−.164*	.236*
QOL2	.371*	.457*	.627*	.479*	.525*	−.214*	−.329*	−.520*	−.194*	−.143*	.245*
QOL3	.334*	.374*	.406*	.636*	.434*	−.191*	−.289*	−.312*	−.491*	−.204*	.217*
QOL4	.258*	.329*	.430*	.371*	.698*	−.095	−.278*	−.267*	−.211*	−.464*	.147*
Pain (VAS, 0–10)	−.052	.032	.024	−.032	.031	.146*	.207*	.147*	.110*	.122*	−.155*
Itch (VAS, 0–10)	.056	−.077	−.021	−.060	−.030	−.112*	−.134*	−.072	−.016	−.060	.065
Number of joints with ROM limit	−.473*	−.215*	−.160*	−.139*	−.146*	.410*	.241*	.246*	.110*	.191*	−.269*
PSQI score	−.305*	−.333*	−.441*	−.400*	−.407*	.161*	.275*	.340*	.171*	.173*	−.163*
SDS score	−.275*	−.291*	−.344*	−.307*	−.347*	.155*	.233*	.320*	.180*	.175*	−.173*
SAS score	−.268*	−.257*	−.338*	−.320*	−.335*	.127*	.247*	.331*	.183*	.207*	−.180*
RTW	.059	.040	.050	.010	.070	−.106*	−.049	−.022	.010	.040	.068
Time after injury onset to rehabilitation	−.117*	−.290*	−.378*	−.275*	−.300*	.041	−.048	−.034	.019	−.081	−.138
Duration of treatment	−.009	.285*	.283*	.202*	.172*	.261*	.430*	.311*	.250*	.260*	−.055

*MBI* Modified Barthel Index, *QOL* World Health Organization Quality of Life BREF scale (WHOQOL-BREF) with four domains including physical health (QOL1), psychological health (QOL2), social relationships (QOL3), and environment (QOL4), *PSQI* Pittsburgh Sleep Quality Index, *SDS* self-rating depression scales, *SAS* self-rating anxiety scales, *RTW* return to work, *VAS* visual analogue scale

*p < 0.05

Table 6 shows the results of the multivariate models fitted to identify independent predictors of MBI and WHOQOL-BREF at discharge and their change during the treatment period and RTW within 6 months after discharge, respectively. For MBI at discharge, the baseline MBI and WHOQOL-BREF scores and the TBSA burned represented the significant predictor variables with *R* ^2^ of 0.463. The prediction equation, [MBI at discharge = 68.9 + 0.297 (MBI at admission) + 0.083 (WHOQOL-BREF for psychological health at admission) − 0.898 (TBSA)], indicates that people with better baseline functional independence and QOL for psychological health and smaller TBSA burned would gain higher functional independence at discharge. For the WHOQOL-BREF scores in physical health and environment at discharge, the significant predictor variables included WHOQOL-BREF in the respective domains at admission, duration of treatment, and timing of intervention. The prediction model indicates that earlier and longer period of rehabilitation and higher baseline QOL in physical health and environment may result in higher WHOQOL-BREF scores in those domains at discharge. For WHOQOL-BREF scores in psychological health and social relationships at discharge, each had an additional predictor: WHOQOL-BREF score in environment for the former and WHOQOL-BREF score in psychological health for the latter, in addition to scores at admission, duration of treatment, and timing of rehabilitation.

**Table 6:** Multivariate predictors of outcomes at discharge

Outcomes	Predictors	*B*	SE	Standardized	95 % CI of *B*	*t* value	*p* value	*R* ^2^
				*B*				
MBI at discharge	Constant	68.873	2.694			25.568	< .001	0.463
	MBI at admission	.297	.021	.585	.255 to .339	13.964	< .001	
	QOL2 at admission	.083	.030	.112	.024 to .141	2.792	.005	
	TBSA burned	−.898	.445	−.086	−1.772 to −.024	−2.020	.044	
QOL1 at discharge	Constant	30.698	1.744			17.605	< .001	0.469
	QOL1 at admission	.478	.030	.573	.418 to .538	15.661	< .001	
	Duration of treatment	.044	.005	.298	.034 to .055	8.189	< .001	
	Onset days	−.019	.085	−.241	−.024 to −.013	−6.622	< .001	
QOL2 at discharge	Constant	26.663	2.168			12.298	< .001	0.510
	QOL2 at admission	.484	.038	.550	.410 to .558	12.815	< .001	
	Duration of treatment	.033	.005	.219	.022 to .043	6.307	< .001	
	Onset days	−.017	.084	−.218	−.023 to −.012	−6.132	< .001	
	QOL4 at admission	.078	.038	.088	.004 to .152	2.073	.039	
QOL3 at discharge	Constant	26.030	2.347			11.091	< .001	0.434
	QOL3 at admission	.458	.043	.494	.373 to .543	10.644	< .001	
	Duration of treatment	.027	.005	.184	.016 to .037	4.923	< .001	
	Onset days	−.011	.089	−.147	−.017 to −.006	−3.837	< .001	
	QOL2 at admission	.116	.040	.135	.038 to .194	2.907	.004	
QOL4 at discharge	Constant	22.916	2.042			11.220	< .001	0.572
	QOL4 at admission	.644	.031	.686	.497 to .644	20.655	< .001	
	Duration of treatment	−.428	.084	−.170	.016 to .036	−5.109	< .001	
	Onset days	.025	.005	.157	−.018 to −.008	4.777	< .001	
Change of MBI	Constant	70.965	3.165			22.421	< .001	0.787
	MBI at admission	−.689	.021	−.901	−.731 to −.647	−32.537	< .001	
	Duration of treatment	.026	.005	.137	.017 to .035	5.697	< .001	
	Payer source	−1.713	.473	−.088	−2.643 to −.784	−3.624	< .001	
	QOL2 at admission	.075	.028	.068	.020 to .131	2.662	.008	
	TBSA burned	−.923	.431	−.059	−1.769 to −.076	−2.142	.033	
	Family support	−1.329	.638	−.048	−2.584 to −.074	−2.082	.038	
Change of QOL1	Constant	30.463	2.342			13.009	< .001	0.474
	QOL1 at admission	−.534	.035	−.611	−.602 to −.466	−15.400	< .001	
	Duration of treatment	.050	.006	.321	.039 to .061	8.772	< .001	
	TBSA burned	−1.345	.519	−.104	−2.365 to −.324	−2.589	.010	
Change of QOL2	Constant	21.311	2.071			10.290	< .001	0.385
	QOL2 at admission	−.485	.039	−.598	−.562 to −.408	−12.418	< .001	
	Duration of treatment	.035	.005	.258	.025 to .046	6.583	< .001	
	QOL4 at admission	.093	.039	.114	.016 to .170	2.369	.018	
Change of QOL3	Constant	22.223	2.162			10.278	< .001	0.321
	QOL3 at admission	−.522	.043	−.606	−.607 to −.437	−12.025	< .001	
	Duration of treatment	.029	.006	.211	.018 to .039	5.181	< .001	
	QOL2 at admission	.131	.040	.164	.052 to .210	3.245	.001	
Change of QOL4	Constant	16.210	2.023			8.014	< .001	0.278
	QOL4 at admission	−.418	.038	−.564	−.493 to −.343	−10.938	< .001	
	Duration of treatment	.028	.005	.223	.018 to .038	5.316	< .001	
	QOL2 at admission	.153	.038	.207	.078 to .228	4.019	< .001	
RTW within 6 months after discharge	*B*	SE	OR	95 % CI of OR	Wald	*P*	.249	
	Age	−.037	.012	.963	.941 to .986	9.523	.002	
	Education	.352	.137	1.422	1.088 to 1.859	6.635	.010	
	Payer source	−.207	.142	.813	.615 to 1.075	2.114	.146	
	TBSA burned	−.799	.123	.450	.354 to .572	42.527	< .001	
	QOL4 at admission	.022	.008	1.022	1.006 to 1.039	7.045	.008	
	Pain (VAS, 0–10)	−.108	.054	.898	.808 to .998	3.961	.047	

All results were processed by multiple linear regression (for MBI and WHOQOL-BREF) and logistic regression analyses (for RTW)

*B* regression coefficient, *SE* standard error, *CI* confidence interval, *MBI* Modified Barthel Index, *QOL* World Health Organization Quality of Life BREF scale (WHOQOL-BREF) with four domains including physical health (QOL1), psychological health (QOL2), social relationships (QOL3), and environment (QOL4), *TBSA* total body surface area,  *PSQI* Pittsburgh Sleep Quality Index, *SDS* self-rating depression scales, *SAS* self-rating anxiety scales, *RTW* return to work, *VAS* visual analogue scale

For the changes in MBI and WHOQOL-BREF during the treatment period, lower baseline values (*r* = −0.418 to 0.688) and longer rehabilitation period (*r* = 0.026 to 0.050) predicted greater changes. In addition, the payer source with higher percentage of expense reimbursed by insurance, higher QOL in psychological health, smaller area of burn, and better family support predicted better improvements in MBI. The change in QOL in physical health can be further predicted by the TBSA burned. The change in QOL in social relationships and environment were both further predicted by QOL in psychological health at admission.

Age, educational level, payer source, TBSA burned, and WHOQOL-BREF score in environment and pain at admission were found to be the significant predictive factors on the success of RTW. The prediction equation remained the same while controlling for the variable of TBSA burned. Older age, larger TBSA burned, and greater pain score were found to be predictors of low RTW rate. In contrast, higher educational level, more health care expense reimbursed, and higher QOL in environment at admission suggested greater probability of returning to work within the 6 months’ follow-up.

## Discussion

This is the first attempt to systematically analyze the RTW, QOL, and functional independence of burn survivors after rehabilitation in mainland China. Results showed that the baseline performance in function and QOL appeared to be strongly related to the functional outcomes. In addition, family support, payer source, early rehabilitation, and duration of rehabilitation intervention appeared to be another group of predictors for QOL and RTW status. The TBSA burned, age, educational level, and level of pain on admission were also found to influence the outcomes of patients.

Higher functional independence at the time of admission to rehabilitation was identified to positively predict improvement in functional independence and higher functional independence at discharge in our study, which is consistent with the findings in previous studies of burn injury in USA populations [4, 5]. The baseline QOL was found to play the same predictive role on the rehabilitation outcome of QOL in people with burn injuries. These results indicate that enhancement of functional independence and QOL in each domain should be emphasized during rehabilitation to promote the patient’s maximum recovery of health.

Payer source was found to predict functional outcomes at discharge in our study, which is, to some extent, inconsistent with that in previous studies [4, 5]. The type of payer source with more expense reimbursed was found to positively predict improvement in functional independence in our study. In contrast, the primary payer resource of Medicare insurance but not the unreimbursed has been found to negatively predict the gain in functional independence in two USA-based studies [4, 5]. In China, there are three main payer sources which include work injury insurance, medical care insurance, and self-payment. The expense reimbursement for the types and duration of rehabilitation therapies are for the most part not limited for a person with work injury insurance. However, although available, these are limited to people with medical care insurance. People without any insurance unfortunately have to pay all expenses out of pocket. Work injury insurance in China covers about 30 % of urban employees, and medical insurance covers only 20 % of the urban population, and these percentages are likely to be smaller if migrant workers were included [21]. Patients with insurance might generally be able to achieve better outcomes because they do not need to worry too much about the cost of rehabilitation. In the USA, the medical insurance covers nearly universally [22], which typically includes Medicare, Medicaid, workers compensation, and commercial insurance. Therefore, the finding of negative impact of Medicare on gain in functional abilities in the previous studies could be secondary to age or pre-injury functional limitation due to disabilities [4, 5].

In the present study, duration of treatment was found to positively predict QOL at discharge and its gains. The mean duration of treatment was 127 days which was much longer than that in the USA (18 to 21 days on average) [3–5]. If sufficient medical service resources and economic support are provided, and without negative impacts on returning to work, extending the duration of treatment could be a good option to achieve better QOL. In addition, earlier rehabilitation was found to predict higher QOL at discharge, although the number of days since burn injury before the start of rehabilitation was much more (157 days) than in the USA (45 days) [4, 5]. The finding concurs with the recommendation of early rehabilitation after injury [23, 24]. However, one should be cautious that missing data could introduce possibility of bias in the present study because different observations might have been resulted if there were no missing data.

The TBSA burned was found to be a negative predictor of functional independence at discharge and its gain during rehabilitation in this study. This finding is inconsistent with that in the previous studies [4, 5]. The present study used MBI as the outcome measure to reflect functional independence, whereas Schneider et al. [4] and Tan et al. [5] used the Functional Independence Measure (FIM). The different outcome measures might explain the discrepant findings pertaining to the effect of TBSA burned in predicting functional independence at discharge. In addition, the TBSA burned negatively predicted RTW, which is consistent with the previous findings as the best predictor of time to RTW [25]. While TBSA burned is a non-modifiable factor, it did not predict the QOL in three domains except for physical health.

In the current study, the likelihood of returning to work within 6 months after discharge was 61 %, which is comparable to other studies conducted in developed countries [18, 19]. In our study, TBSA burned and the QOL at admission, age, educational level, payer source, and pain condition were found to be predictors of RTW. This result remained the same while controlling for the factor of TBSA burned, which increased the rigor of the predictive roles of these variables. Our results are consistent with a previous study that demonstrated the predictive role of TBSA burned, QOL, and bodily pain on RTW [18, 19, 26]. However, unlike our results, the study by Dyster-Aas et al. [26] found no predictive relationship between age and educational level on return of work. The mean age of the burn injury subjects in their study was 41 years, which is greater than that of our subjects (35 years). Also, the average educational level of the subjects in that Swedish study was high school (24 %) and above [26], but our subjects had mostly high school (36 %) or lower (53 %) level education. This should be noted since the educational level and age could determine the type of work a person engages in. It is possible that the types of work for people with higher educational level and older age are more knowledge based or skill based, which do not significantly impede them from returning to work after injury. Therefore, the type of work may be a key predictor of RTW, and future studies should examine this factor. In addition, the prediction of payer source on RTW in the present study may be related to the positive effects of a higher reimbursement rate, similar to the effects on QOL outcomes.

Despite meaningful findings on prediction of rehabilitation outcomes in a population with burn injuries in China, the present study has some limitations. First, missing data could introduce possible bias because different observations might have been resulted if there were no missing data. The payer source and short duration of treatment could be the causes of missing data, which showed a difference in results with that in the cases included. Second, although the facility where all data were collected is the rehabilitation center for burn injury in mainland China, and the categories of cases could be representative to some extent, the reader should still be cautious about the generalizability of the study outcomes to the whole population in mainland China with burn injury. Third, the reliability and validity of the measurements of rehabilitation outcomes used in people with different disabilities have been reported by a number of previous studies [11, 12]; however, the use of these measurements in burn patients have not been examined in this study. Further studies examining the reliability and validity of these measurements in burn patients in mainland China are needed to strengthen the credibility of the findings. Fourth, the present study only included the burn patients admitted to the rehabilitation hospital; thus, the results of this study may not be predictive for the rehabilitation outcomes of the acute surgery patients. Fifth, the lack of objective definitions, possible ambiguity, or subjectivity of the terms “family support” and “economic status” is also a limitation of this study. Sixth, as individuals’ discharge dates are variable, the final measure (6 months post-discharge) from the rehabilitation hospital is a moveable time point, so some patients might have more time to recover than others, which was not accounted for in the analyses. Another limitation of the present study is the lack of information related to the type of work of the patients, which likely confounds the interpretation of the role of educational level and age on predicting RTW. The type of work which the patients engage in should be recorded in future studies in order to explore its role on predicting RTW in burn patients in China.

In addition to the above limitations, we would like the reader to be cautious of the following when interpreting the results of the present study. First, this study is an assessment of the performance of a single rehabilitation facility in China which admits and treats a casemix with a majority of workplace injury survivors. Second, the collection and analysis of specific time-related data was not planned a priori, which might influence the interpretation of QOL and RTW outcomes as these are related to discharge from the rehabilitation facility, not the date of injury. Furthermore, the influence of payer source (workplace injury) might introduce significant bias and must be considered in the generalization of results. Finally, work-related injury patients receive more in-patient care and rehabilitation staff input and have greater duration of access to in-patient rehabilitation services than other patients included in this study. These might all contribute an impact on the results of the present study.

## Conclusions

To conclude, higher baseline functional independence and QOL, payer source with higher percentage of expense reimbursed, better family support, earlier and longer duration of rehabilitation, younger age, higher educational level, lower bodily pain, and smaller area of burn predicted better rehabilitation outcome in burn injury patients in mainland China. Identification of predictors may help assess the rehabilitation potential of burn survivors and assist resource allocation. Among these variables, higher baseline self-care performance and QOL, as well as earlier and longer duration of rehabilitation are modifiable factors, and we suggest policy makers to allocate sufficient medical resources to ensure them.

## Abbreviations

FIM, Functional Independence Measure; MBI, Modified Barthel Index; PSQI, Pittsburgh Sleep Quality Index; QOL, quality of life; RTW, return to work; SAS, self-rating anxiety scale; SDS, self-rating depression scale; TBSA, total body surface area; VAS, visual analogue scale; WHO, World Health Organization
